# Psychotic‐like experiences associated with sleep disturbance and brain volumes in youth: Findings from the adolescent brain cognitive development study

**DOI:** 10.1002/jcv2.12055

**Published:** 2021-12-02

**Authors:** Jessica R. Lunsford‐Avery, Katherine S. F. Damme, Teresa Vargas, Maggie M. Sweitzer, Vijay A. Mittal

**Affiliations:** ^1^ Department of Psychiatry and Behavioral Sciences Duke University School of Medicine Durham North Carolina USA; ^2^ Department of Psychology Northwestern University Evanston Illinois USA; ^3^ Institute for Innovations in Developmental Sciences Northwestern University Evanston and Chicago Illinois USA; ^4^ Department of Psychiatry Northwestern University Chicago Illinois USA; ^5^ Department of Medical Social Sciences Northwestern University Chicago Illinois USA; ^6^ Institute for Policy Research Northwestern University Chicago Illinois USA

**Keywords:** brain volumes, psychosis, psychotic‐like experiences, sleep, structural MRI, thalamus

## Abstract

**Background:**

Sleep disturbance is characteristic of schizophrenia and at‐risk populations, suggesting a possible etiological role in psychosis. Biological mechanisms underlying associations between sleep and psychosis vulnerability are unclear, although reduced sleep‐regulatory brain structure volumes are a proposed contributor. This study is the first to examine relationships between psychotic‐like experiences (PLEs; subclinical symptoms reflecting psychosis vulnerability/risk), sleep, and brain volumes in youth.

**Methods:**

Brain volumes of five sleep‐related structures were examined in relation to PLEs and difficulties initiating and maintaining sleep (DIMS) in 9,260 9‐ to 11‐year‐olds participating in the Adolescent Brain Cognitive Development (ABCD) study. Analytic models examined relationships between DIMS, PLEs, and brain volumes, as well as DIMS as a mediator of brain volume–PLEs relationships. Although sleep regulation structures (i.e., thalamus, basal forebrain, and hypothalamus) were of primary interest, other potentially relevant structures to sleep‐related functioning and psychosis (i.e., hippocampus and amygdala) were also examined.

**Results:**

PLEs were associated with increased DIMS as well as reduced volume in some, but not all, brain structures, including the thalamus and basal forebrain in children. DIMS was also associated with reduced left thalamus volume in youth. Increased DIMS partially, statistically mediated the relationship between left thalamic volume and PLEs, although the effect was relatively small.

**Conclusions:**

Results highlight left thalamic volume as a potential neural mechanism underlying sleep disturbances and PLEs in childhood. Future studies should assess causal relationships between sleep, PLEs, and brain structure across adolescent development, interactions with other psychosis risk factors, and the role of sleep interventions in prevention of psychosis and a range of psychiatric conditions across the lifespan.

## Graphical abstract

Sleep disturbances are associated with psychotic‐like experiences (PLEs) in the general population, yet biological mechanisms underlying sleep and psychosis vulnerability remain unclear. This study is the first to example relationships between PLEs, sleep, and structural brain volumes in a large representative sample of youth, and found that the relationship between left thalamus volume, a key structure in sleep regulation and PLEs was partially, statistically mediated by increased difficulties initiating and maintaining sleep. Results highlight a potential role of sleep and sleep‐regulatory brain structures in psychosis etiology and suggest a possible prevention target during early development.

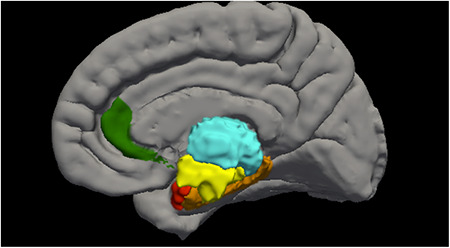

**Key points**

- Sleep disturbances are common in psychosis and at‐risk populations, and reduced sleep‐regulatory brain volumes represent possible neural contributors
- This study investigates relationships between psychotic‐like experiences (PLEs), sleep, and sleep‐related brain volumes in preadolescence, prior to the primary developmental window of psychosis vulnerability
- Poor sleep was related to PLEs in youth, and a specific sleep‐regulatory brain volume—the left thalamus—was associated with both sleep and PLEs
- Sleep disturbance partially, statistically mediated the association between reduced left thalamic volume and PLEs, although the effect was small
- Results highlight a potential role of sleep and sleep‐regulatory brain structures in psychosis etiology and suggest a possible prevention target during early development

## INTRODUCTION

Sleep and psychosis are integrally intertwined. Sleep disturbances, including difficulties initiating and maintaining sleep (DIMS), have long been observed as a key feature of schizophrenia associated with positive and negative symptoms, distress, and functional impairment (Waite et al., 2020). Additionally, growing evidence suggests that poor sleep precedes psychosis onset, generating interest in a possible role of sleep in its etiology and pathophysiology (Davies et al., 2017; Lunsford‐Avery & Mittal, 2013; Reeve et al., 2015). Studies also suggest that sleep disturbance may be related to psychotic‐like experiences (PLEs; Barton et al., 2018; Davies et al., 2017; Reeve et al., 2015; Waite et al., 2020), which reflect elevated vulnerability as well as risk for psychosis in the general population (Kelleher & Cannon, 2011). Specifically, sleep and PLEs have been linked in population/community (DeVylder & Kelleher, 2016; Jeppesen et al., 2015; Koopman‐Verhoeff et al., 2019; Koyanagi & Stickley, 2015; Lee et al., 2012; Taylor et al., 2015), clinical (Cosgrave et al., 2018), and college student samples (Andorko et al., 2017; Ered et al., 2018; Simor et al., 2019). Indeed, cognitive‐behavioral therapy for insomnia has been shown to reduce psychotic experiences in young adults, suggesting insomnia may play a causal role in PLEs (Freeman et al., 2017).

Although the precise contribution of sleep to psychosis pathogenesis remains unclear, potential mechanisms include links between neuromaturation, cognition, and biological and social stress with sleep during adolescence—a critical period in which biological and environmental interactions may drive psychosis onset (Lunsford‐Avery & Mittal, 2013). Elucidating mechanisms underlying poor sleep and its relationship with PLEs early in development is critical for understanding a continuum of psychosis vulnerability and for advancing efficacious prevention and intervention efforts.

PLEs are subclinical psychosis symptoms (e.g., unusual beliefs and abnormal perceptual experiences) experienced by a subset of the general population (Kelleher & Cannon, 2011). They occur early in development (13%–15% of children; Laurens et al., 2007; Poulton et al., 2000). Common risk factors underlie pediatric PLEs and psychosis, including cognitive deficits, internalizing symptoms, and family history of psychosis (Karcher et al., 2018), and experiencing childhood PLEs confers risk for psychosis onset (Kelleher et al., 2012; Poulton et al., 2000; Welham et al., 2009). Because PLEs fall along a phenotypic continuum with clinical psychosis, examination of links between PLEs and sleep in the general youth population may clarify potential mechanisms underlying sleep and its associations with psychosis etiology. Additionally, investigating these relationships in childhood may illuminate risk markers early in development, prior to psychosis onset and during a window in which identification and prevention may be particularly critical. However, is important to note that PLEs do not always develop into psychosis (Sullivan et al., 2020), and indeed, are also associated with a risk of a range of psychiatric disorders beyond psychosis, including affective, anxiety, behavioral, and substance‐use disorders (Healy et al., 2019; Trotta et al., 2020), suggesting clarifying links with sleep may have implications for broader mental health.

As mentioned above, altered neural development is one hypothesized mechanism underlying poorer sleep, and perhaps in turn, broader psychosis symptomology (Lunsford‐Avery & Mittal, 2013). Human sleep is regulated by a complex brain system including both arousal‐ and sleep‐promoting regions, including the thalamus, hypothalamus, basal forebrain, brainstem, and cortex (Schwartz & Kilduff, 2015). Abnormalities in some of these areas, such as the thalamus, are consistently observed in schizophrenia (Shepherd et al., 2012; van Erp et al., 2016). The thalamus, through its reciprocal interplay with the cortex, plays key roles in initiating both wakefulness and non‐rapid eye movement (NREM) cortical rhythms, such as sleep spindles, which may in turn impact the onset, stabilization, and termination of sleep (Gent et al., 2018). Reduced thalamic volume is observed early in the course of schizophrenia and continues to decline over time (Dietsche et al., 2017). Deficits in thalamic‐initiated sleep oscillations have been proposed as a psychosis biomarker (Steullet, 2019), highlighting its potential importance in linking sleep and psychosis. Reductions of other sleep‐regulatory regions (e.g., hypothalamus, basal forebrain) have also been observed, but less frequently, in schizophrenia (e.g., for review see Lunsford‐Avery & Mittal, 2013).

Notably, our prior work has shown reduced bilateral thalamic volume among adolescents at clinical‐high risk (CHR) for psychosis (Lunsford‐Avery et al., 2013), which is consistent with other studies suggesting reduced gray matter of the thalamus and/or its subregions in genetic high risk or CHR samples (Steullet, 2019). Moreover, results suggested that reduced thalamic volume was associated with greater self‐reported difficulties with initiating and maintaining sleep (i.e., increased sleep latency and decreased efficiency and quality; Lunsford‐Avery et al., 2013). These findings suggest that thalamic structural abnormalities may clarify an underlying neural vulnerability contributing to problematic sleep among adolescents vulnerable to psychosis. However, whether this relationship exists across the psychosis spectrum, is present *prior* to the primary window of psychosis vulnerability (i.e., adolescence), and extends to other sleep‐regulatory brain regions is unknown.

Emerging in the last decade, a body of research now supports a relationship between sleep disturbances and PLEs (Barton et al., 2018; Davies et al., 2017; Reeve et al., 2015; Waite et al., 2020). This link has been observed globally (Koyanagi & Stickley, 2015), is present in childhood (Jeppesen et al., 2015; Koopman‐Verhoeff et al., 2019) and adolescence (Lee et al., 2012; Taylor et al., 2015), and is associated with PLE‐related distress (Andorko et al., 2017) and poorer functioning (DeVylder & Kelleher, 2016). Although a range of sleep problems have been examined, difficulties with initiating and maintaining sleep (e.g., fragmented sleep, reduced duration, and/or poor perceived quality) and insomnia symptoms have been most frequently associated with PLEs across the lifespan (Andorko et al., 2017; Cosgrave et al., 2018; Jeppesen et al., 2015; Koopman‐Verhoeff et al., 2019; Lee et al., 2012). Notably, one study has shown that poor sleep quality predicts increased PLEs, but not the reverse (Simor et al., 2019), supporting a possible causal role of sleep in PLEs (Barton et al., 2018).

The Adolescent Behavior Cognitive Development (ABCD) study (Barch et al., 2018; Garavan et al., 2018) offers an unprecedented opportunity to examine brain mechanisms linking sleep and PLEs in a general population of preadolescents (Karcher & Barch, 2020). Prior studies using this sample have shown that PLEs can be reliably assessed in children (Karcher et al., 2018) and share overlapping neural (Karcher et al., 2019) and environmental (Karcher et al., 2020) correlates with PLEs, supporting validation of the PLE construct across the lifespan. In addition, parent‐reported sleep duration and/or disturbances have been examined in the ABCD sample in relation to brain structure, overall psychiatric health (e.g., depression), and cognition (Cheng et al., 2020; Goldstone et al., 2020). Notably for the current study, one investigation suggested an association between shortened sleep duration and reduced thalamic volume (Cheng et al., 2020).

The current study had several objectives. First, we examined relationships between brain structures and (1) PLEs and (2) DIMS in the ABCD sample. Based on the broader schizophrenia literature, as well as our prior work in CHR adolescents, we were primarily interested in associations between *sleep‐regulatory* volumes (specifically the thalamus, as well as the basal forebrain and hypothalamus; Scammell et al., 2017; Schwartz & Kilduff, 2015) and these clinical phenomena. Additionally, to examine the specificity of relationships, we examined other regions implicated in *sleep‐related functioning* and psychosis (hippocampus and amygdala; Ganzola et al., 2014; Haukvik et al., 2018; Ho et al., 2019; van Erp et al., 2016). Although less integral to sleep regulation, the hippocampus and amygdala are central to sleep‐related emotional and cognitive processing, including sleep‐dependent memory consolidation (Marshall et al., 2020). Second, we investigated the relationship between PLEs and DIMS in the ABCD sample. Finally, if significant associations were found for the first two objectives, we examined whether a relationship between the structural difference in sleep‐related brain region(s) and PLEs in children was mediated by DIMS. If mediation effects are present, it may suggest that both sleep disturbances and PLEs partially arise out of abnormal development of specific brain regions, and that there may be a possible downstream effect of sleep problems which increase the risk of PLEs.

## METHODS

### Participants

ABCD includes more than 11,000 children aged 9–11 years from 21 centers across the United States, achieving a range of demographic diversity (Barch et al., 2018; Garavan et al., 2018). This analysis focuses on assessments conducted at participants’ baseline evaluation. In cases in which there were siblings who completed the study, one child per family was randomly selected for inclusion, resulting in the exclusion of 1,809 potential participants. Group demeaning per site accounted for possible nesting effects within sites per recommendations (Bear et al., 2011; Huang, 2016; Huang & Cornell, 2016), and analyses were conducted with site demeaned values for PLEs, DIMS, and brain volumes. Parents’ written informed consent and children's assent were obtained. Research procedures were in accordance with the ethical guidelines laid out by respective Institutional Review Boards (DOI: 10.15154/1519065).

### Clinical assessments

#### Psychotic‐like experiences

PLEs were assessed using the 21‐item, self‐reported Prodromal Questionnaire‐Brief Child Version (Loewy et al., 2011), which has adequate internal reliability and construct validity (e.g., associations with other PLE measures, such as psychosis family history) in the ABCD sample (Karcher et al., 2018). The questionnaire asked children about specific PLEs which were endorsed with a binary response (yes/no). Total scores were calculated as the total number of endorsed symptoms, and range from 0 to 21; higher total scores indicate more PLEs endorsed. Importantly, this scale does not inquire about PLEs occurring in the context of sleep/wake initiation.

Although our primary analyses focused on PLE total score as to assess the relationship between DIMS and a population‐based continuum of PLEs, we explored associations between DIMS and PLEs associated with distress, as distress may indicate clinical significance, as well as between DIMS and specific types of PLEs (i.e., thought delusions, unusual or grandiose delusions, and hallucinations), in supplementary analyses. Regarding distress, for each PLE symptom endorsement, participants indicated whether there was distress related to the symptom on a 5‐point Likert scale. As with prior research (Karcher et al., 2018; Loewy et al., 2011), a total PLE with distress score was calculated by summing the total number of endorsed symptoms weighted by level of distress (0 = no endorsement, 1 = endorsement, no distress, 2–6 indicate endorsement with incremental levels of distress). Regarding specific PLE factors, we drew from prior research (Azis et al., 2021) to categorize symptoms into three factors: thought delusions (questions 1, 5, 8, 12, 14, and 18), unusual or grandiose delusions (questions 4, 7, 15, and 16), and hallucinations (questions 2, 3, 9, 10, 11, 17, 19, and 20); three questions (6, 13, and 21) were not included in this analysis as it is not clear to which factor they should be assigned.

#### Sleep Disturbance Scale for Children (SDSC)

The parent‐reported SDSC assesses a range of sleep disorders of childhood (e.g., sleep breathing, insomnia, sleep‐wake transition, and excessive somnolence; Bruni et al., 1996). As these disorders are heterogeneous in terms of presentation and etiology, the current analyses focused specifically on the 7‐item *DIMS* subscale, which characterizes the frequency of specific difficulties in initiating (e.g., “The child has difficulty getting to sleep at night”) and maintaining sleep (e.g., “After waking up in the night, the child has difficulty to fall asleep again”) on a Likert‐type scale ranging from “Never” (1) to “Always” (5). This subscale was selected for its relevance to psychosis, in which insomnia symptoms are common (Waite et al., 2020), and because it most closely aligned with aspects of sleep previously linked to thalamic volumes in CHR youth (Lunsford‐Avery et al., 2013). However, to explore the specificity of the relationship of PLEs with DIMS, we examined the relationship of PLE total score to additional SDSC subscales (i.e., SDB, sleep breathing disorders; DA, disorders of arousal, SWTD, sleep‐wake transition disorder; DOES, disorders of excessive somnolence; SHY, sleep hyperhidrosis) in a supplementary analysis.

#### Kiddie Schedule for Affective Disorders and Schizophrenia for School‐Age Children, Present and Lifetime Version (K‐SADS‐PL)

The K‐SADS‐PL is a semi‐structured parent‐child interview that evaluates for the presence of a range of psychiatric disorders (Kaufman et al., 1997). Consistent with prior ABCD studies (Pagliaccio et al., 2020), the total count of current depression symptoms indicated as present based on child report, here calculated without sleep items, was included as a continuous measure of depression symptoms and covariate in analyses. Similarly, the total count of mania symptoms indicated as present based on child report, again calculated without the sleep item, was included as a continuous measure of mania symptoms and covariate in analyses.

### Magnetic resonance imaging (MRI) acquisition and processing

**FIGURE 1:**
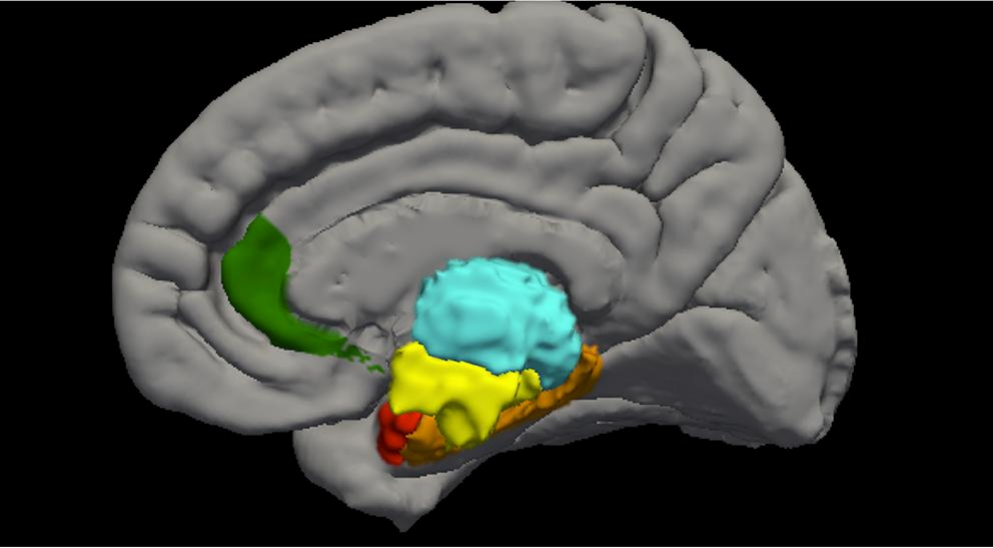
Regions of interest in the current study, including thalamus (teal), hypothalamus (yellow), basal forebrain (green), hippocampus (orange), and amygdala (red)

Gray matter volume data were accessed from the Annual Curated Data Release 2.01 from the ABCD consortium (https://abcdstudy.org/index.html). A total of 11,533 children completed MRI scan sessions at 21 centers on a variety of scanners, including GE Healthcare, Philips Healthcare, and Siemens Healthcare. These scan sessions included a high‐resolution *T*1‐weighted structural MRI scan (1‐mm isotropic voxels), and all structural data were processed with FreeSurfer version 5.3.0 (http://surfer.nmr.mgh.harvard.edu/; Dale et al., 1999; Fischl et al., 1999) using processing steps that have been published and described (Casey et al., 2018). Additional participant and methodological details are available at the ABCD website (https://abcdstudy.org/scientists/protocols/). Quality control for structural images comprised visual inspection of *T*1 images and FreeSurfer outputs for quality conducted by the ABCD team. Participants for whom a failing rating was given during inspection (due to severe artifacts or irregularities) were excluded from the current analyses. Morphometric measures consisting of gray matter volume as defined by the Desikan‐Killiany Atlas were accessed from the national data archive from the ABCD study release (Hagler et al., 2019). Volumes were selected based on their relationship to sleep disturbances and/or psychosis: including thalamus, hypothalamus (i.e., ventral dorsal column), basal forebrain (i.e., rostral anterior cingulate cortex), hippocampus, and amygdala (Figure 1).

Volume was chosen for several reasons. First, volume reflects a composite of surface area and thickness (Wierenga et al., 2014; Winkler et al., 2018). Additionally, we did not have specific surface area or thickness‐related hypotheses; therefore, volume allows for a broader insight into both area and thickness without increasing the number of model parameters. Second, using volume enhances ease of incorporating our findings into extant literature that has also depended on volumes (Shepherd et al., 2012; van Erp et al., 2016). Of the initial sample, 464 total participants were excluded for either failing to meet the quality control criteria of the ABCD team or for not containing the full information of both the sleep measures and the structural images. Participants with both DIMS and brain volumes available totaled 11,069 youth.

### Analytical strategy

First, linear regressions examined relationships between each brain volume of interest (thalamus, basal forebrain, hypothalamus, amygdala, and hippocampus) separately by left and right hemisphere and (1) PLE and (2) DIMS total scores. Given that the distribution of PLE total scores was non‐normal, we applied a BoxCox transformation with lambda values chosen to maximize the loglikelihood for a normal distribution (Osborne, 2010). Prior to applying the BoxCox transformation, the data were shifted so that all values were positive. All analyses reported in the results use the transformed variable. Given multiple comparisons (20 models), a Bonferroni correction was applied, with significance set at *p* < .0025. Second, linear regression examined the PLEs‐DIMS relationship. Finally, volume(s) shown to be related to both DIMS and PLEs in the earlier aims were evaluated in separate mediation analyses. These mediation analyses tested the significance of the indirect effect of relevant brain volume(s) on PLEs via DIMS. Analyses were conducted using bootstrapping (5,000) with bias‐corrected and accelerated (BCa) 99% confidence intervals using PROCESS v3.5 implemented in SPSS version 27 (Hayes, 2018). For comparison, we also tested an alternative mediational model, in which we examined whether a relationship between DIMS and PLEs in children was mediated by the structural difference in sleep‐related brain region(s). Using analogous procedures as those described above, these analyses tested the significance of the indirect effect of DIMS on PLEs via the relevant brain volume(s).

Covariates for all analyses were selected in order to be consistent with a prior study examining sleep, brain volumes, and psychiatric health in ABCD (Cheng et al., 2020), and as such, to allow the current paper to be integrated into the larger neurodevelopmental sleep literature. Covariates were entered in Step 1 of each linear regression, and included age, sex, body mass index, puberty score, race, and parents’ income and number of years of education. Additionally, we covaried for current depression and mania symptoms in Step 2. Predictors of interest were entered in Step 3. Figures were created using R (R Core Team, 2019).

Supplementary analyses using partial Pearson correlations and the covariates described above explored the specificity of the PLE–sleep relationships reported in the primary analyses by examining associations between (1) DIMS and other PLE‐related variables (PLE total including distress and separately by PLE factor) and (2) between PLE total score and other sleep disturbances (SDB, DA, SWTD, DOES, and SHY).

## RESULTS

### Participants

Analyses included 9,260 participants aged 9–11 (see Table 1).

**TABLE 1:** Sample demographics

Variable (*n* = 9260)	Mean	*SD*
Age	9.90	0.62
Parental education (years)	16.37	2.71
Pubertal Development Scale	8.03	2.41

	Number	Percentage
Sex (female)	4,399	48
Race/ethnicity		
African American/Black	1,385	15
Hispanic/Latinx	1,735	19
White	4,865	53
Multiracial	960	10
Other	315	3
Household income		
<$5,000	331	4
$5,000–11,999	339	4
$12,000–15,999	227	3
$16,000–24,999	406	4
$25,000–34,999	532	6
$35,000–49,999	722	8
$50,000–74,000	1,162	13
$75,000–99,999	1,240	13
$100,000–199,999	2,544	28
$200,000+	960	10
Not reported	797	9

### Brain volumes related to PLEs

#### Thalamus

**FIGURE 2:**
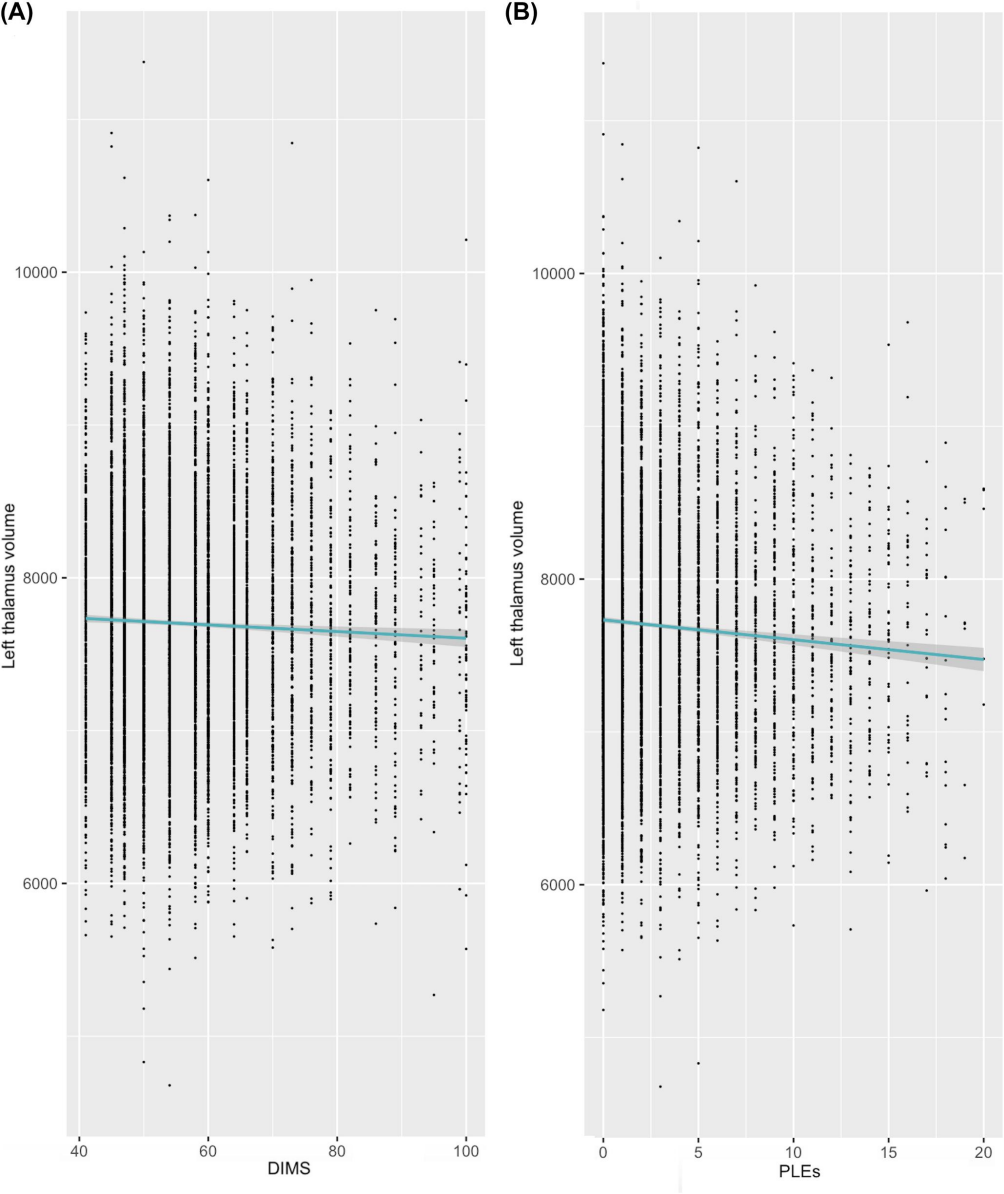
Associations of the left thalamus to (A) difficulties initiating and maintaining sleep (t‐score) and (B) psychotic‐like experiences. For ease of interpretation, data is shown prior to site demeaning and BoxCox transformation

After covarying for demographics in step 1 (*F* (10,7881) = 24.85, *p* < .001) and mood symptoms in step 2 (*F* (2,7879) = 205.47, *p* < .001, *partial‐r* for depression = .05, *partial r* for mania = *.22*), reduced left (*F* (1,7878) = 11.89, *p* < .001, *partial r* = −.04), but not right (*F* (1,7878) = 9.13, *p* = .003, *partial r* = −.03), thalamic volume was associated with increased PLE total score (see Figure 2).

#### Basal forebrain

After covarying for demographics and mood symptoms, reduced left (*F* (1,7878) = 18.90, *p* < .001, *partial r* = −.05) volume of the basal forebrain was associated with elevated PLE total. The relationship between right basal forebrain volume and PLEs was not significant (*F* (1,7878) = 3.32, *p* = .07, *partial r* = −.02).

#### Hypothalamus

Neither the left (*F* (1,7878) = 5.44, *p* = .02, *partial r* = −.03) nor the right (*F* (1,7878) = 3.88, *p* < .05, *partial r* = −.02) hypothalamus volumes were related to elevated PLE total score after covarying for demographics and mood symptoms.

#### Amygdala

Neither amygdala volume in the left (*F*(1,7878) = 5.63, *p* = .02, *partial r* = −.03) nor the right (*F*(1,7878) = 7.96, *p* < .005, *partial r* = −.03) hemispheres were associated with greater PLE total after adjustment of multiple comparisons.

#### Hippocampus

Neither left (*F* (1,7878) = .43, *p* = .51, *partial r* = −.01) nor right (*F* (1,7878) = .01, *p* = .93, *partial r* = .001) hippocampal volume were related to PLE total.

### Brain volumes related to DIMS

#### Thalamus

After covarying for demographics in step 1 (*F* (10,7880) = 7.31, *p* < .001) and mood symptoms in step 2 (*F* (2,7878) = 116.38, *p* < .001, *partial‐r* for depression = .15, *partial‐r* for mania = .07), decreased left thalamic volume, *F* (1,7877) = 11.71, *p* < .001, *partial r* = −.04, but not right thalamic volume, *F* (1,7877) = 6.16, *p* = .01, *partial r* = −.03, was related to increased DIMS (see Figure 2).

#### Basal forebrain

After controlling for demographics and mood symptoms, the relationship of left basal forebrain volume and DIMS did not survive correction for multiple comparisons, *F* (1,7877) = 5.82, *p* = .02, *partial r* = −.03. There was no significant relationship between right basal forebrain volume and DIMS, *F* (1,7877) = 3.10, *p* < .10, *partial r* = −.02.

#### Hypothalamus

Relationships between left, *F* (1,7877) = 6.96, *p* = .008, *partial r* = −.03, and right, *F* (1,7877) = 5.32, *p* = .02, *partial r* = −.03) hypothalamus volume and DIMS also did not survive correction for multiple comparisons after controlling for demographics and mood symptoms.

#### Amygdala

Neither left amygdala volume, *F* (1,7877) = .59, *p* = .44, *partial r* = −.01, nor right amygdala volume, *F* (1,7877) = .33, *p* = .57, *partial r* = −.01, were associated with DIMS after accounting for covariates.

#### Hippocampus

Neither left, *F* (1,7877) = 3.69, *p* = .06, *partial r* = −.02, nor right, *F* (1,7877) = 1.57, *p* = .21, *partial r* = −.01, hippocampus volume was associated with DIMS.

### DIMS related to PLEs

**FIGURE 3:**
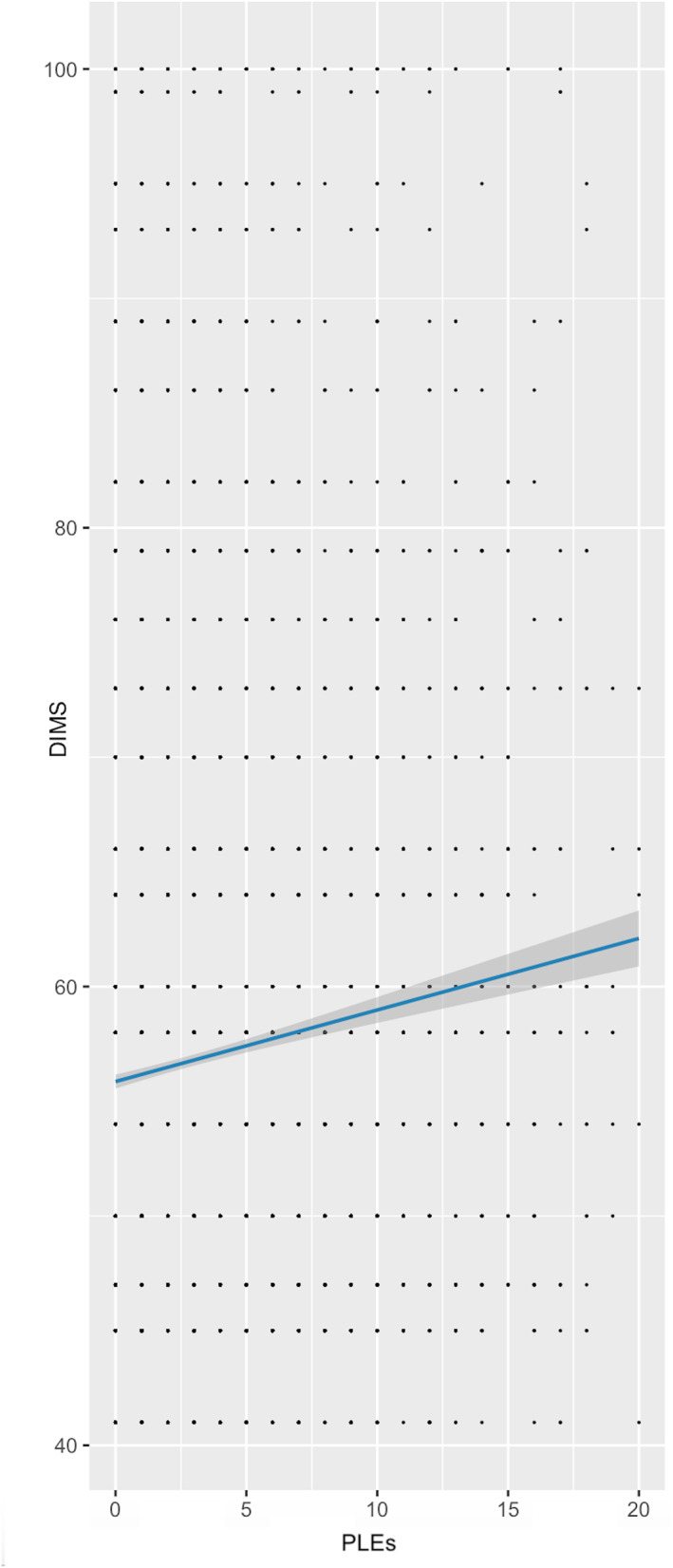
Relationship between difficulties initiating and maintaining sleep (t‐score) and psychotic‐like experiences. For ease of interpretation, data is shown prior to site demeaning and BoxCox transformation

After covarying for demographics in step 1 (*F* (10,7880) = 24.84, *p* < .001) and mood symptoms in step 2 (*F* (2,7878) = 205.44, *p* < .001, *partial‐r* for depression = .05 and *partial‐r* for mania = .22), increased DIMS total score was related to increased total PLEs, *F* (1,7877) = 71.76, *p* < .001, *partial‐r* = .10 (Figure 3).

### Follow‐up mediation analysis

**FIGURE 4:**
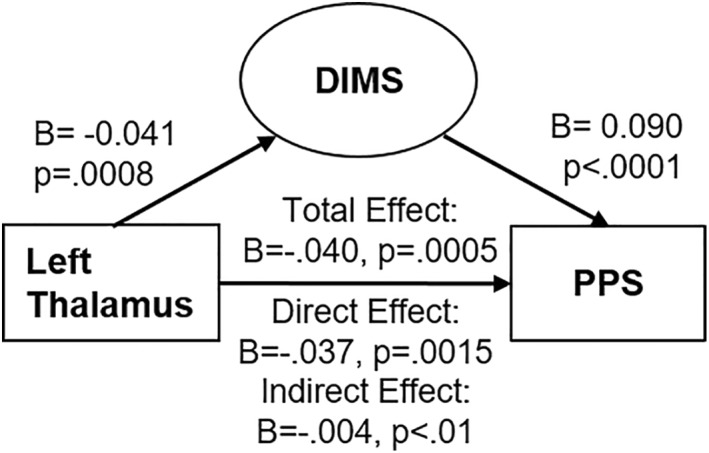
Direct and indirect effects of left thalamic volume on psychotic‐like experiences, mediated by difficulties initiating and maintaining sleep. Coefficients are reported as unstandardized values, adjusted for multiple covariates included in the model. *p*‐Value reported for indirect effect reflects zero falling outside of 99% confidence interval

Given that the left thalamus volume was associated with both DIMS and PLEs, a mediation analysis examined if DIMS statistically mediated the relationship between left thalamic volume and PLEs. DIMS significantly mediated the association between left thalamic volume and PLEs (*B* = −0.0036; BCa 99% CI: −0.0069 to −0.0009; Figure 4 and Table S1). Of note, this indirect effect accounted for ∼9% of the total effect of thalamic brain volume on PLEs. In the alternative mediational model, left thalamic volume also significantly mediated the association between DIMS and PLEs (*B* = 0.0013; BCa 99% CI: 0.0001 to 0.0031), and this indirect effect accounted for ∼1% of the total effect of DIMS on PLEs (see Figure S1).

### Supplemental analyses

Exploratory analyses regarding additional considerations related to PLEs suggested that a total PLE score that incorporated distress ratings, as well as each PLE factor (hallucinations, unusual or grandiose delusions, and thought delusions) were related to DIMS total score, and that effect sizes were similar to that observed using the PLE total score reported above (see Table S2). Exploratory analyses regarding other sleep disturbances suggested that symptoms of other sleep disorders, including sleep‐wake transition disorders and disorders of excessive somnolence, were related to PLE total score, while sleep breathing disorders, disorders of arousal, and sleep hyperhidrosis were less strongly associated (Table S3).

## DISCUSSION

Building on prior literature showing sleep disturbances and gray matter reductions in psychosis and high risk populations, as well as a growing number of studies showing links between PLEs and sleep disturbance, the current study examined relationships between youth brain structures, sleep, and PLEs. Although sleep‐regulatory volumes were primary structures of interest, additional structures relating to sleep‐related functioning and psychosis were examined to assess the specificity of sleep‐regulatory brain volumes to the sleep‐PLEs relationship. Results suggested that reduced sleep‐regulatory brain volumes, including the thalamus and basal forebrain, were correlated with PLEs in youth, but only the left thalamus was related to DIMS. Additional structures commonly related to psychosis and more peripherally related to sleep, such as the amygdala and hippocampus, were uncorrelated with either clinical construct after Bonferroni correction. Notably, DIMS significantly mediated the relationship between left thalamic volume and PLEs, suggesting that abnormalities in thalamic development may partially underlie both sleep disturbances and PLEs. Additionally, sleep problems arising from thalamic abnormalities may have a downstream impact on increasing PLEs. Notably, an alternative mediational model found that left thalamic volume significantly mediated the relationships between DIMS and PLEs, providing additional support for links between left thalamic volume, sleep, and PLEs; however, the indirect effect accounted for a smaller portion of the total effect in the alternative model. Regardless, it is important to note that statistical mediation does not imply causation, as longitudinal research is needed to address that question.

These findings are interesting for several reasons. First, the last 10 years have seen a surge of interest in a possible etiological role of sleep in psychosis. The current study adds to a growing literature suggesting that sleep disturbances are observed across the psychosis continuum and early in development, prior to the modal age of psychosis onset (Davies et al., 2017; Lunsford‐Avery & Mittal, 2013; Reeve et al., 2015), and that identifying sleep disturbances, particularly those occurring in conjunction with PLEs, in childhood may offer avenues for early intervention. Short‐term behavioral treatments (Dewald‐Kaufmann et al., 2019), have recently been shown to reduce common symptom correlates of PLEs, including internalizing symptoms in youth (Lunsford‐Avery et al., 2021) as well as positive psychosis symptoms in CHR adolescents (Bradley et al., 2018) and PLEs in young adults (Freeman et al., 2017). Future studies may evaluate the utility of behavioral sleep strategies in reducing PLEs in childhood as well as attenuating psychosis risk in later life. In addition, as noted above, PLEs are not only associated with psychosis risk, but risk for a range of psychiatric conditions, from mood/anxiety disorders to behavioral and substance‐use disorders (Healy et al., 2019; Trotta et al., 2020), and future studies should examine the impact of sleep interventions on improving broader psychiatric health associated with PLEs.

Second, this study highlights one specific neural mechanism which may partially underlie the link between sleep disturbances and psychosis; that is, reduced left thalamic volume. This finding is consistent with our prior study showing associations between reduced thalamic volume and increased sleep latency, and reduced efficiency and quality, among CHR youth (Lunsford‐Avery et al., 2013) as well as a broader literature showing thalamic reductions in schizophrenia (Shepherd et al., 2012; van Erp et al., 2016) and at risk populations (Steullet, 2019). It is important to note, however, that although the effect of sleep on PLEs was partially mediated by left thalamic volume, the effect was relatively small. A relatively small effect is likely observed for several reasons. First, a neurodevelopmental model hypothesizes a range of mechanisms linking sleep and psychosis pathogenesis, of which associations with neurodevelopment is only one. It is likely that sleep‐related neurodevelopmental factors interact with other risk factors impacted by sleep, including underlying genetic and biological vulnerabilities, cognition, and environmental stress, as well as other non‐sleep related factors, to drive psychosis onset (Lunsford‐Avery & Mittal, 2013). In addition, the neurobiology of both sleep (Scammell et al., 2017; Schwartz & Kilduff, 2015) and psychosis (Ganzola et al., 2014; Haukvik et al., 2018; Ho et al., 2019; van Erp et al., 2016) is highly complex, and additional risk factors not examined by the current study may also play a role. However, given this complexity, it is particularly notable that this study suggests that thalamic volume, and in the left hemisphere specifically, appears to be key in underlying links between sleep to psychosis. It is also important to note that additional brain regions not examined in the current study, such as the ventral tegmental area and regions of the cortex, may also play a role in sleep and psychosis, and should be the focus of further study.

This study has several limitations. First, this cross‐sectional analysis does not allow for causal inferences and elucidating the direction of the observed effects is an important target for future studies. Given the longitudinal design of the ABCD study, the current study may serve as a baseline for observing how relationships between brain volumes, sleep, and PLEs change and influence each other over the course of adolescent development. In particular, studies following youth into late adolescence and young adulthood—a key developmental period for psychosis risk as well as risk for other psychiatric disorders associated with PLEs (e.g., mood disorders)—will be informative for clarifying the role of thalamic abnormalities and sleep disturbances in psychiatric risk. Second, this study relies on a parental‐report of sleep rather than objective measures, such as polysomnography. Research has suggested that deficits in sleep spindles, or 12–16 Hz NREM sleep oscillations generated within the thalamus, are characteristic of schizophrenia, including first‐episode and high risk populations, and may contribute to the cognitive deficits seen in this population (Ferrarelli & Tononi, 2017; Manoach & Stickgold, 2019). Future work may shed light on the role of objectively measured, thalamic‐driven sleep indices, such as sleep spindles, on PLEs and cognition in youth.

## CONCLUSION

This study is the first to examine relationships between brain volumes, sleep disturbances, and PLEs in a general population of youth, and the first to show that an association between reduced left thalamic volume and pediatric PLEs is partially, statistically mediated by sleep disturbance. Results highlight a potential role of sleep and a sleep‐regulatory brain structure in psychosis etiology, and as sleep is a modifiable behavior, suggest a possible prevention target during early development. Future studies may examine how sleep and structural brain abnormalities interact with other risk factors across adolescent development to confer psychosis risk later in life as well as risk for the range of disorders associated with PLEs.

## CONFLICT OF INTERESTS

The authors declare that they have no competing or potential conflicts of interest.

## AUTHOR CONTRIBUTIONS

Drs. Lunsford–Avery and Mittal conceptualized the study and developed the methodology with input from Dr. Damme, and Dr. Lunsford–Avery led the investigation. Drs. Lunsford–Avery and Mittal acquired funding. Drs. Damme, Vargas, and Lunsford–Avery contributed to data curation, and Drs. Damme, Lunsford–Avery, and Sweitzer developed the analysis code and completed formal analyses. Drs. Damme, Vargas, and Sweitzer created the visualizations of the presented data. Dr. Lunsford–Avery prepared the original draft of the manuscript with input from Dr. Mittal. Drs. Mittal, Damme, Sweitzer, and Vargas critically reviewed and edited the manuscript. All authors checked and approved the final version of the manuscript.

## ETHICAL CONSIDERATIONS

Parents’ written informed consent and children’s assent were obtained. Research procedures were in accordance with the ethical guidelines laid out by respective Institutional Review Boards (doi: 10.15154/1519065).

## Supporting information

Supporting Information S1

## Data Availability

The data that support the findings of this study are openly available as part of the Adolescent Brain Cognitive Development (ABCD) Study (https://abcdstudy.org), which is stored in the NIMH Data Archive (NDA).
