# Supplementary material for: Psychotic‐like experiences associated with sleep disturbance and brain volumes in youth: Findings from the adolescent brain cognitive development study
Source: JCPP Adv. 2021 Dec 2;1(4):e12055. doi: 10.1002/jcv2.12055 (PMC9635573; doi:10.1002/jcv2.12055)

Supplemental Table 1. Total, Direct, and Indirect Effects for Mediation Models, with 99% Confidence Intervals

| Effect (of Left Thalamic Volume on PLE Total) | Parameter Estimate (B) | Confidence Interval | % Total Effect |
| --- | --- | --- | --- |
| Total Effect | -.0402 | [-.070; -.010] | 100% |
| Direct Effect | -.0366 | [-.066; -.007] | 91.0% |
| Indirect Effect (through DIMS) | -.0036 | [-.007; -.001] | 9.0% |

Abbreviations: PLE, Psychotic-Like Experiences; DIMS, Difficulty Initiating and Maintaining Sleep

Supplemental Table 2. Partial Correlations between DIMS and PLE Factors and Total PLE Score including Distress

| Additional PLE-Related Variables | DIMS |
| --- | --- |
| PLE Factors |  |
| Unusual/Grandiose Delusions | 0.05*** |
| Thought Delusions | 0.07*** |
| Hallucinations | 0.10*** |
| PLE Total Including Distress Ratings | 0.10*** |

Note: All variables account are mean-centered by site to account for nesting. Partial correlations control for age, BMI, pubertal development, family income, parental education, sex, race, and current depression and mania symptoms. Significance: ***p<.001.

Abbreviations: DIMS, Difficulty Initiating and Maintaining Sleep; PLE, Psychotic-Like Experiences.

Supplemental Table 3: Partial Correlations between Additional Sleep Indices and PLE Total Score

| Sleep Variable | PLE Total |
| --- | --- |
| SBD | .02* |
| DA | .03* |
| SWTD | .05*** |
| DOES | .06*** |
| SHY | .02 |

Note: All variables account are mean-centered by site to account for nesting. PLE total reflects a BoxCox transformed variable. Partial correlations control for age, BMI, pubertal development, family income, parental education, sex, race, and current depression and mania symptoms. Significance: ***p<.005, **p<.01, *p<.01.

Abbreviations: SDB, sleep breathing disorders; DA, disorders of arousal, SWTD, sleep-wake transition disorder; DOES, disorders of excessive somnolence; SHY, sleep hyperhidrosis; PLE, Psychotic-Like Experiences.

Supplemental Figure 1. Direct and indirect effects of DIMS on PLEs, mediated by left thalamic volume. Coefficients are reported as unstandardized values, adjusted for multiple covariates included in the model. P value reported for indirect effect reflects zero falling outside of 99% confidence interval.


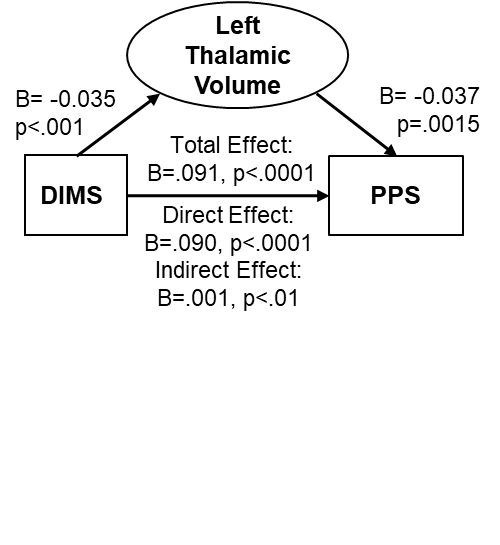

Supplement: Supplementary file 1 — Supporting Information S1 [file JCV2-1-e12055-s001.docx]
